# A New Type of Hierarchical Honeycomb in-Plane Impact Study

**DOI:** 10.3390/ma14081917

**Published:** 2021-04-12

**Authors:** Hang Song, Chenyang Zhang, Pengyu Wang, Lingzhi Meng, Zhenqing Wang

**Affiliations:** College of Aerospace and Civil Engineering, Harbin Engineering University, Harbin 150001, China; 15738864067@163.com (H.S.); 13070118516@163.com (C.Z.); pengyu_wang@hrbeu.edu.cn (P.W.); mlz95828@163.com (L.M.)

**Keywords:** hierarchical honeycomb, failure stress, impact, finite element

## Abstract

Honeycomb materials have low density, high specific strength and stiffness, impact resistance, and good sound insulation effect, and therefore are widely used in aerospace, automobile, and ship field applications. In this paper, we study the in-plane impact response of a second-order hierarchical honeycomb (SHH) material. Its main structure is a hexagonal honeycomb, and the substructure is composed of an augmented double arrow honeycomb (ADAH) negative Poisson’s ratio unit. Through a finite element simulation, the failure stress of an hierarchical honeycomb in two directions of quasi-static crushing and dynamic crushing was analyzed; the failure stress of the hierarchical honeycomb under different densities, different speeds, and different substructures was discussed; and the theoretical failure stress was verified. The numerical analysis results show that a second-order hierarchical honeycomb (SHH) has better collapse stress than a first-order regular hexagonal honeycomb (FHH) and an augmented double arrow honeycomb (ADAH).

## 1. Introduction

Honeycomb materials have good mechanical properties and excellent thermal conductivity, therefore, they are widely used in aerospace, construction, transportation, and other fields [1,2,3,4,5]. Additionally, there are many multicellular cell structures in nature, such as wood, bone, and the outer skin of plant cells that are light weight and have good mechanical properties, and therefore they have been the focus of many bionic studies [6,7,8,9]. Honeycomb materials without classification are called first-order honeycomb materials. First-order honeycomb materials have greater specific strength and specific stiffness than traditional materials and have excellent application prospects in energy absorption. First-order honeycomb materials mainly include square, diamond, hexagon, triangle, and kagome shapes [3,10,11,12,13,].

With the continuous development of honeycomb materials, a class of metamaterials with negative Poisson’s ratio units has appeared. This class of metamaterials does not refer to artificially manufactured materials; it is a physical property, not a material property and it realizes the negative Poisson’s ratio effect through its own structural properties. This class of metamaterial can shrink after being compressed and has high fracture toughness [14], as well as excellent anti-explosion performance [15,16,17]. Its indentation hardness is also very high [18]. Some studies have focused on double arrow honeycomb and concave hexagonal honeycomb, and a series of related formulas have been deduced and verified [19,20,21].

With the development of industrial engineering, ordinary honeycomb materials do not meet the requirements of specific stiffness and strength, and therefore hierarchical honeycomb materials have emerged. The mechanical properties of hierarchical honeycomb materials are better than those of single-level honeycomb materials. However, due to the technical limitations of the manufacturing industry, most of the hierarchical honeycomb materials only exist in theory, and the existing manmade materials can only achieve a few hierarchical levels. In order to promote the engineering application of hierarchical honeycomb materials, Jinxiu Qiao and Changqing Chen analyzed the hierarchical honeycomb formed by replacing hexagonal cell walls with equilateral triangular substructures. They deduced the theoretical value of the failure stress from the deformation mode, verified and analyzed it with a finite element, and analyzed the influence of both different impact velocities and the number of substructures on the failure stress [22]. Additional studies on hierarchical honeycombs have added substructures such as squares, hexagons, and kagomes [23,24,25,26,27,28]. H. L. Tan et al. added triangular subunits into concave hexagons and obtained excellent mechanical properties [29,30].

However, when these honeycomb structures [22,23,24,25,26,27,28] are subjected to quasi-static impact, the strain corresponding to the linear elastic zone is relatively large and cannot reach the platform zone quickly, therefore, the energy absorption effect is poor in the early stage. In addition, when the structure is impacted, the stress–strain curve in the platform area oscillates violently, which weakens the reliability of the theoretical and numerical results. In order to solve these problems, in this study, we embed augmented double arrow honeycomb (ADAH) with negative Poisson’s ratio effect as a substructure into a first-order regular hexagonal honeycomb (FHH) to form a new type of hierarchical honeycomb, which we call a second-order hierarchical honeycomb (SHH) Using a combined theory and numerical method, we verify that the SHH has better collapse stress than either the first-order regular hexagonal honeycomb (FHH) or the augmented double arrow honeycomb (ADAH).

**Figure 1:**
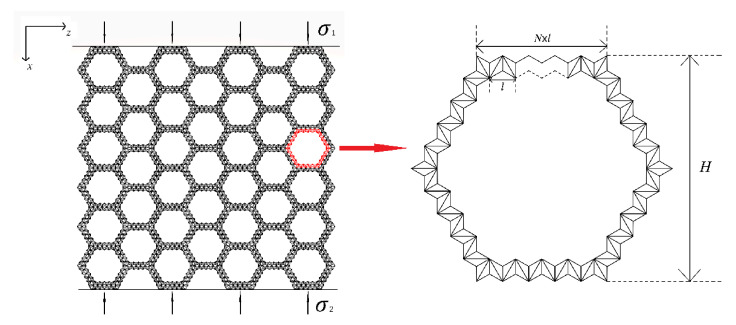
Schematic diagram of a second-order hierarchical honeycomb SHH and cells under impact load in the x direction.

This paper is organized as follows: In the second section, we introduce the material and structure of the layered honeycomb and the research methods used. In the third section, we use the finite element method to study the collapse response of the layered honeycomb structure in the x and z directions under quasi-static uniaxial compression (the x and z directions are shown in Figure 1). The analytical expression of collapse stress is derived based on the simulated deformation mode. We also study the dynamic impact resistance of the layered honeycomb structure under impact, as well as analyze the error between the finite element results and the formula fitting results of the dynamic impact resistance of structures with different numbers of substructures. In the fourth section, we state our conclusions.

## 2. Materials and Methods

Due to the development of industrial engineering, there are higher requirements for modern materials, which are, first, to have excellent mechanical properties, and secondly, to be economical. Aluminum has excellent mechanical properties and a low cost, and therefore meets the requirements of modern industrial development. Therefore, we chose aluminum for the modeling. When determining the mechanical model, it is important that the selected mechanical model must conform to the actual situation of the material, because only in this way can the calculation results reflect the real stress and stress state in the structure or structures. At the same time, the mathematical expressions of the mechanical model should not be too complicated to avoid mathematical difficulties in solving problems. We compared the stress–strain curve of aluminum and found that it was roughly the same as the stress–strain curve of an ideal elastoplastic material. Therefore, in this study, we set the aluminum material as an ideal elastoplastic material.

Indeed, all the materials used in this study were ideal plastic materials. We set the density of the material as $\rho_{s}=2700\;kg/m^{3}$, the modulus of elasticity as E=70 GPa, the yield stress as $\sigma_{s}=110\;MPa$, and the Poisson’s ratio as $\nu_{s}=0.3$. For a better illustration, when analyzing the SHH, we established an x-z coordinate system, where the x and z directions are shown in Figure 1.

### 2.1. Theoretical Method of Second-Order Hierarchical Honeycomb (SHH) Static Failure Stress during Impact in the x and z Directions

According to the deformation pattern diagram, an analytical model of the SHH structure collapse stress can be established. Here, we used the two-scale method to derive the analysis results, that is, the failure analysis of the whole unit under the two scales of macro-deformation failure and substructure failure, and then the theoretical equation was obtained. This method uses the failure of the substructure to cause the instability of the overall structure to deduce the collapse stress of the SHH structure.

### 2.2. Theoretical Method of SHH Dynamic Failure Stress during Impact in the x and z Directions

When studying the dynamic failure of the SHH, a certain velocity gradient was established, and the impact model diagrams at different velocities were obtained, and then the dynamic failure stress was obtained according to the law of conservation of momentum [21]. Finally, the theoretical analysis of dynamic failure stress was verified according to the results of finite element analysis.

## 3. Results and Discussion

### 3.1. Second-Order Hierarchical Honeycomb (SHH) and Finite Element Modeling

**Figure 2:**
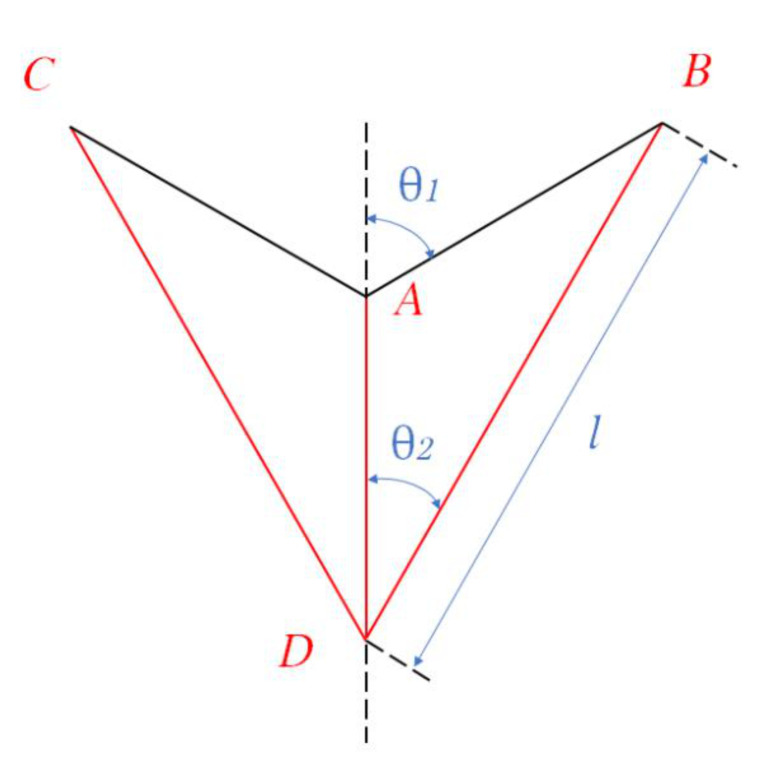
Schematic diagram of the augmented double arrow honeycomb (ADAH) structure.

As described above, hierarchical honeycombs have better mechanical properties than traditional honeycombs. Here, we systematically study the impact response of a second-order hierarchical honeycomb (SHH). Figure 1 shows the schematic diagram of the uniaxial compression of SHH. One end of the SHH is fixed on a rigid plate, and the other end is impacted by another rigid plate. The stress at the impact end is denoted by $\sigma_{1}$ and the stress at the distal end is denoted by $\sigma_{2}$. The cell wall of SHH is composed of a standard augmented double arrow honeycomb (ADAH). The structure of ADAH is shown in Figure 2, where l shows the length of BD in ADAH element, N×l shows the length of the macrostructure edge, and H shows the length of the macrostructure structure. The thickness of the SHH cell wall is h, and the out-of-plane width is b. Assuming that the cell wall of the honeycomb structure is equally distributed, then, the relative density of the SHH is (In this paper, $\theta_{1}$ and $\theta_{2}$ are constants unless otherwise specified. We set $\theta_{1}$ of ADAH structure to π/3 and the $\theta_{2}$ to π/6) as follows:

(1) $$\bar{\rho }=\frac{\left(1+\sqrt{3}\right)\left(8N-4\right)}{\sqrt{3}N^{2}}\frac{h}{l}$$

We used Abaqus/CAE 6.14-4 software to perform the numerical simulation of the SHH, the finite element (FE) model adopts a structure of four macro elements in the z direction and six macro elements in the x direction. The cell wall is simulated by shell elements with five integration points. The unit type is set to S4R which is a universal shell element type in Abaqus that has the following properties: a four-node curved shell element that can be used to model thin or thick shell structures, a reduced integration method that includes hourglass mode control and allows limited membrane strain. For the SHH model, we set l=0.01 m and b=0.02 m.

**Figure 3:**
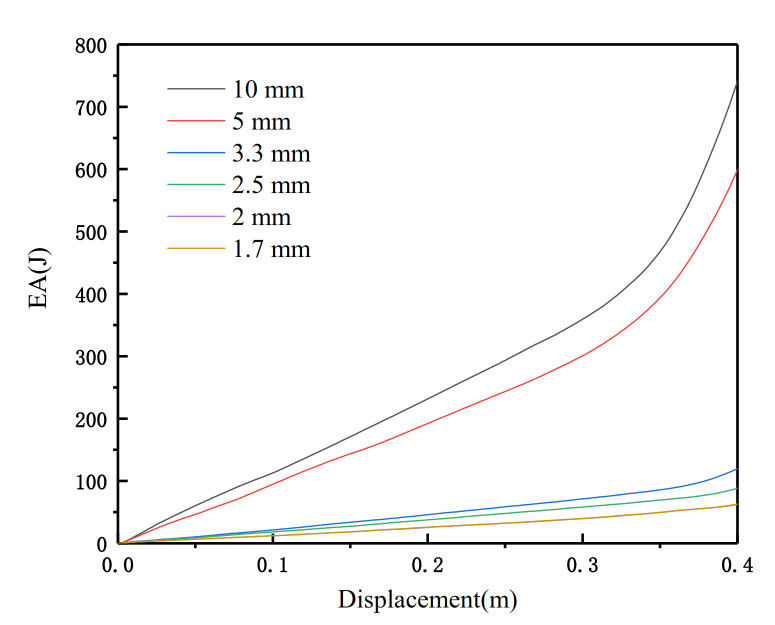
Convergence analysis of element size.

In order to ensure the accuracy of the simulation, we conducted a convergence test on the SHH and determined the appropriate mesh element size of the finite element model. As shown in the figure, we simulated and compared the energy absorption (EA)-displacement curves under different element sizes. It can be seen in Figure 3 that when the shell element size is reduced to 1.7 mm, the EA gradually converges. Therefore, the unit size of 1.7 mm is suitable for modeling these structures. This size is used in the following modeling grid unless otherwise specified. The general contact assumption can be used for the entire model. There is no friction in the tangential direction of the model, and the normal contact behavior is a “hard” contact.

### 3.2. Quasi-Static Collapse

#### 3.2.1. Uniaxial Compression in the x Direction

**Figure 4:**
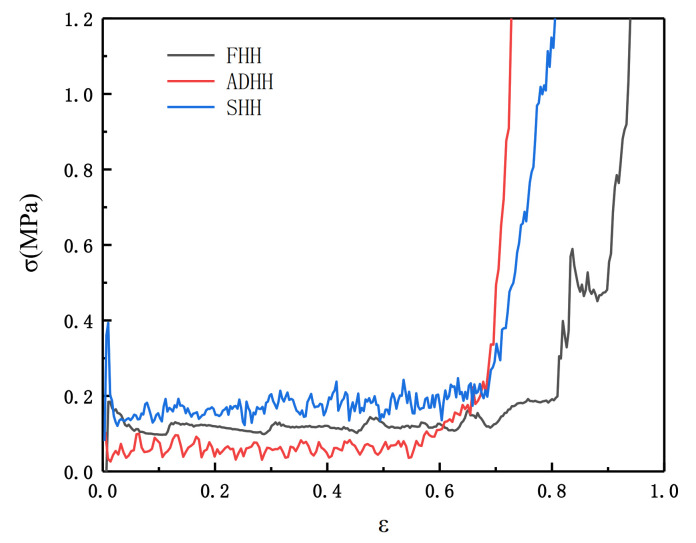
Finite element (FE) predicting the stress map of quasi-static collapse, the N of the second-order hierarchical honeycomb (SHH) equals 5 and $\bar{\rho }=5\%$ in the SHH, the first-order regular hexagonal honeycomb (FHH), and the ADAH.

In this section, we analyze the uniaxial compression of the SHH unit in the x direction. The experiment proves that speed 1 m/s is sufficient to cause a quasi-static collapse, $\bar{\rho }=5\%$. The results of the numerical simulation are shown by the blue line segment in Figure 4. There is an obvious oscillation zone in the SHH because the structure is a membrane-oriented deformation mode, similar to that in the study by X.M. Qiu et al. [10].

In order to form a comparison, we also performed the finite element modeling of the SHH cell wall unit ADAH and the traditional honeycomb FHH. When simulating the ADAH unit, we set the unit structure 9×8 (that is, there are nine elements in the x direction and eight elements in the z direction). For the FHH, we use the structure model of 6×4 (that is, there are nine elements in the x direction and eight elements in the z direction). The number of integrals along the cell wall and the number of out-of-plane integrals are the same as those of SHH. For the ADAH and FHH, the unit size is set to 1.7 mm to ensure the accuracy of the value, which is same as the unit size of the SHH. The out-of-plane displacements of all the honeycomb structures are fixed. The contact mode of the FHH and ADAH is the same as that of the SHH. The result is shown in Figure 4. The red line represents the ADAH and the black line represents the FHH. It can be clearly seen from Figure 4 that the honeycomb unit stress–strain diagram can be divided into three stages, namely the linear elastic zone, the platform zone, and the densification zone. We define the average stress of the platform zone as the collapse stress, which is an important index of energy absorption performance. It can be seen from Figure 4 that the collapse stress of SHH is significantly higher than that of FHH and ADAH. The images of SHH and ADAH have obvious oscillations, while the images of FHH are very smooth. This is because both SHH and ADAH are deformation dominated by film, and FHH is deformation dominated by bending. ADAH entered the densification zone earlier than FHH and SHH. It can be seen that the SHH has greater collapse stress than that of the FHH and ADAH, therefore, SHH has better energy absorption effect, that is, SHH has better performance than that of FHH and ADAH.

It is not an easy task to obtain $\varepsilon_{d}$ of the platform area. Under ideal conditions, the compressive strain $\varepsilon_{d}$ in quasi-static crushing equals $1-\bar{\rho }$, but in fact, there are many deviations. As an empirical equation, we use the equation $\varepsilon_{d}=0.8\left(1-\bar{\rho }\right)$ adopted by Jinxiu Qiao and Changqing Chen [22]. Densification strain $\varepsilon_{d}$ is an important index for the final study of dynamic failure stress, which is introduced in the following sections.

#### 3.2.2. Collapse Model of SHH

**Figure 5:**
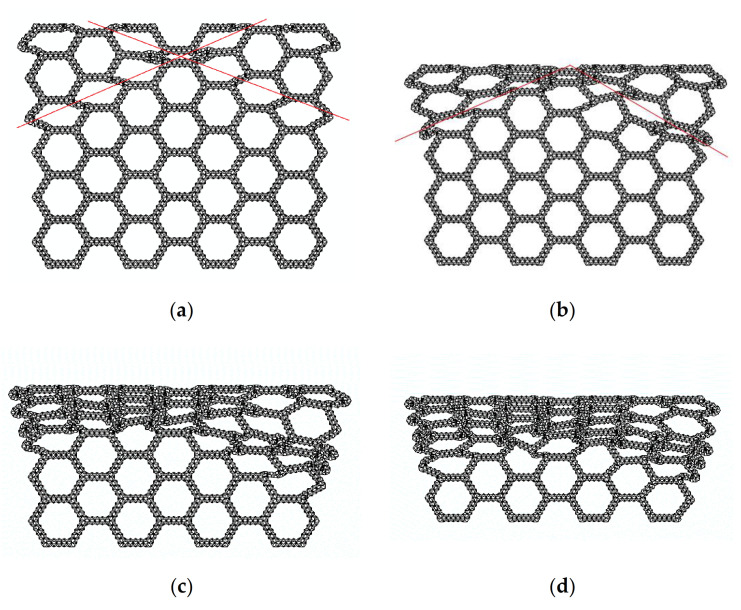
FE prediction of quasi-static compression in the x direction, N=5 and $\bar{\rho }=5\%$. (**a**) ε=12%; (**b**) ε=24%; (**c**) ε=42%; (**d**) ε=50%.

Figure 5 is a series of deformation diagrams of compression in the x direction of the SHH.

In Figure 5a, when the crushing strain ε reaches about 12%, local deformation characteristics of “X” begin to form. This is because the macroscopic regular hexagon in the middle is symmetrically compressed when the deformation starts, and the hexagon elements on both sides begin to slip, which leads to the deformation feature of “X” formed locally. A continuation of compression, as shown in Figure 5b, begins to compact the upper end of the SHH, and when the crushing strain ε reaches 24%, the “X” structure begins to transform into a “V” structure. The SHH continues to deform, as shown in Figure 5c, and when the crushing strain reaches 42%, the first stage of deformation begins to finish. In Figure 5d, the second stage of deformation starts when the crushing strain ε reaches 50%. Please refer to the supplementary materials for the specific deformation mode.

#### 3.2.3. Augmented Double Arrow Honeycomb’s (ADAH’s) FE Model

Before proceeding to the next section, we must first derive the failure stress of the ADAH under uniaxial compression in the z direction (the derivation result is Equation (8)).

**Figure 6:**
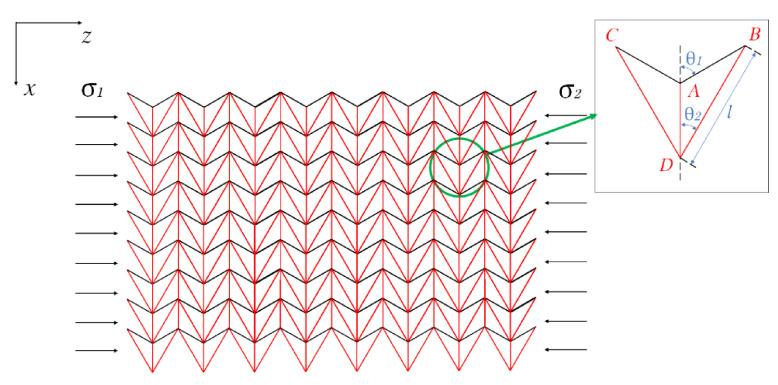
Schematic diagram of the ADAH and cells under impact load in the z direction.

The augmented double arrow honeycomb (ADAH) is shown in Figure 6, which can be expressed by five parameters: $\theta_{1}$, which is the angle between the short side AB and axis x; $\theta_{2}$, which is the angle between the long side BD and axis x; the length of the long side BD which is l; the thickness of the beam which is h; and the out-of-plane width which is b. In this study, let $\theta_{1}$ be π/3 and $\theta_{2}$ be π/6. According to the geometric structure, the relative density of ADAH can be derived as follows:

(2) $$\bar{\rho_{ADAH}}=\frac{\rho_{ADAH}}{\rho_{s}}=\frac{\left(4\sqrt{3}+6\right)}{\sqrt{3}}\frac{h}{l}$$

where $\rho_{s}$ is the density of the base material and the structure size, material, mesh, and contact are the same as that in the finite element simulation in Section 3.1.

#### 3.2.4. Theoretical Model of the ADAH

**Figure 7:**
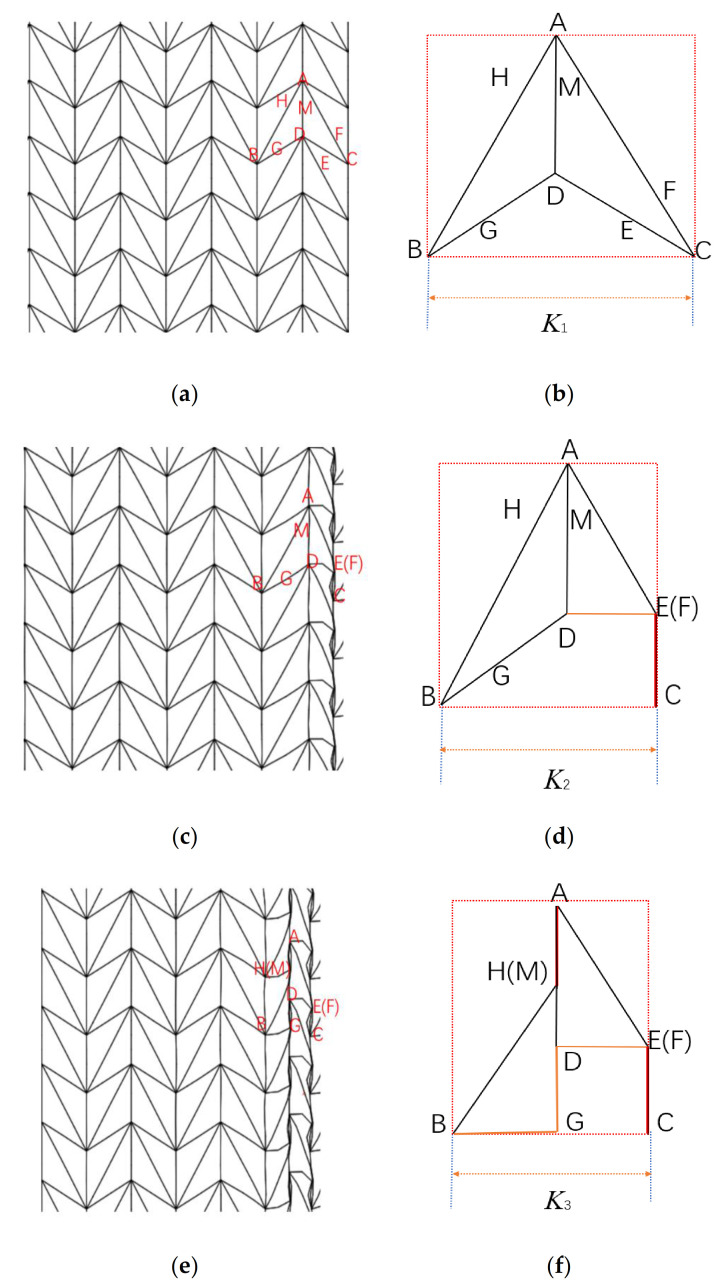
Schematic diagram of the FE prediction of ADAH deformation. (**a**) $t=t_{0}$; (**b**) $t=t_{1}$; (**c**) $t=t_{2}$; (**d**) $t=t_{0}$; (**e**) $t=t_{1}$; (**f**) $t=t_{2}$.

Figure 7 shows the deformation characteristics of the ADAH under the impact of a rigid plate. As shown in Figure 7a,d, at time $t_{0}$, the distance between point B and point C is $K_{1}$. As the rigid plate moves, first, it touches the edge point C, and then the midpoint of DC begins to bend, and the other half of ADAH’s speed is still zero. At time $t_{1}$, as shown in Figure 7b,e, point F and point E coincide (point E is the midpoint of the DC side), and the DE side is perpendicular to the EC side. At this time, the distance between point B and point C is $K_{2}$. The rigid board continues to move, and then the speed of the triangle ABD reaches the impact speed. Due to the action of the next unit, the H and G points begin to bend. At the moment of $t_{2}$, as shown in Figure 7c,f, the point H coincides with the point M, and the BG side is perpendicular to the DG side. At this time, the distance between point B and point C is $K_{3}$, the deformation of the entire unit ends, and the next unit deformation cycle begins. According to the deformation principle, the effective height of the representative unit between time $t_{0}$ and $t_{2}$ can be expressed as follows:

(3) $$\Delta K=2L\sin\theta_{2}-\frac{L\sin\theta_{2}}{\sin\theta_{1}}$$

The total rotating hinge of the hinge formed by eight plastic hinges is as follows:

(4) $$\Delta \theta =2\pi +2\theta_{2}$$

The total work done by the external force is as follows:

(5) $$W=\frac{\sin(\theta_{1}-\theta_{2})\sin\theta_{2}{Lb\sigma }_{0}}{\sin\theta_{1}\sin\theta_{2}}$$

According to the plastic dissipation theory, the plastic loss of the work done by the external force at the hinge is as follows:

(6) $$W=2\Delta {\theta M}_{p}$$

where $M_{p}$, which is the plastic bending moment, can be calculated as follows:

(7) $$M_{p}=\frac{{bh}^{2}}{4}\sigma_{ys}$$

Therefore, the quasi-static failure stress $\sigma_{0}$ can be obtained as:

(8) $$\sigma_{0}=\frac{(\pi +\theta_{2})\sin\theta_{1}{}^{2}}{\sin(\theta_{1}-\theta_{2})(2\sin\theta_{1}\sin\theta_{2}-\sin\theta_{2})}{\left(\frac{h}{l}\right)}^{2}\sigma_{ys}$$

#### 3.2.5. Comparison of Theoretical Analysis of the ADAH with the Finite Element Simulation

**Figure 8:**
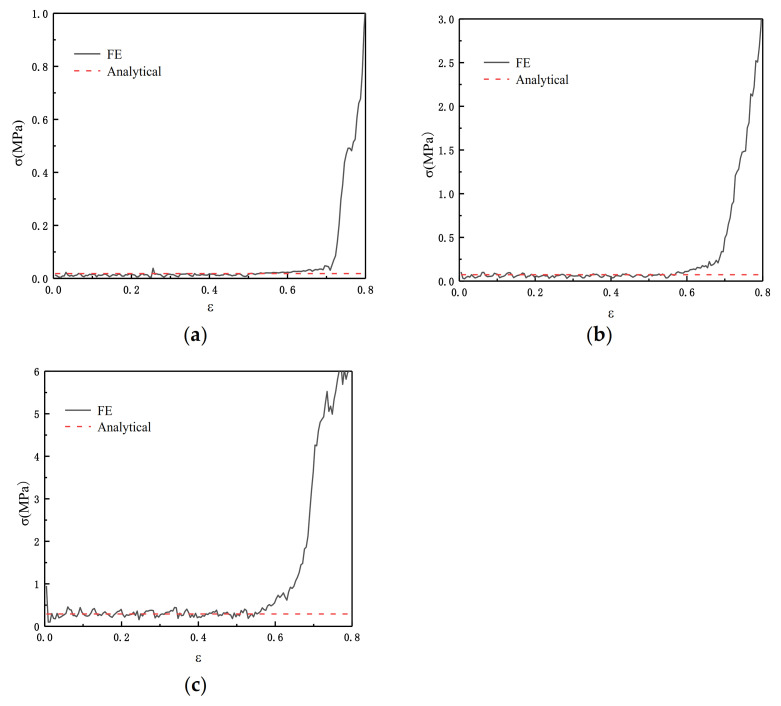
FE predicting ADAH quasi-static (V=1 m/s). (**a**) $\bar{\rho }=2.5\%$; (**b**) $\bar{\rho }=5\%$; (**c**) $\bar{\rho }=10\%$.

In order to verify the results from Section 3.2.4, we substituted the parameters we obtained into the finite element and we set $\bar{\rho }=2.5\%$, $\bar{\rho }=5\%$, and $\bar{\rho }=10\%$. Figure 8 shows the theoretical values and the results of the finite element analysis. The results show that the collapse response is effectively predicted by Equation (8) in Section 3.2.4.

#### 3.2.6. The Failure Stress of the SHH Structure

**Figure 9:**
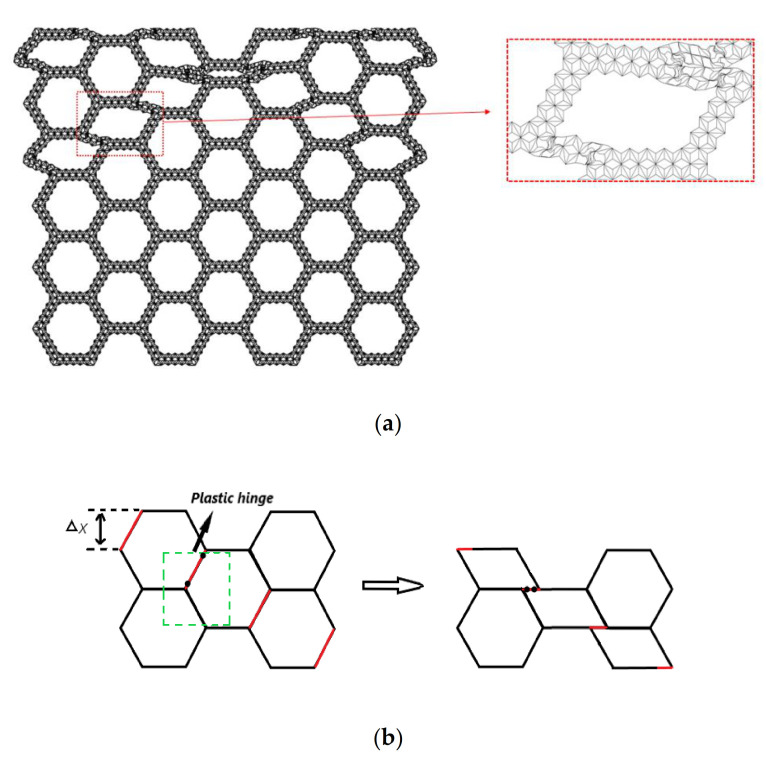
(**a**) Enlarged SHH deformation diagram, N=5, ε=12%, and $\bar{\rho }=5\%$; (**b**) deformation diagram of SHH macrostructure impact deformation in the x direction.

According to the deformation pattern diagram, an analytical model of the collapse stress of the SHH structure can be established. Figure 5 shows the deformation characteristics of the honeycomb structure. We enlarge Figure 5 to obtain Figure 9a. Here, we followed the two-scale method to derive the analysis. In this method, the collapse stress of the SHH structure is derived from the instability of the whole structure caused by the failure of the substructure. This is the reason why we analyzed the damage structure of the ADAH unit in the previous section. Because this method has a wide range of application and high accuracy, it is adopted by a large number of researchers.

As shown in Figure 9b, first, we consider the macroscopic deformation. In the hexagon, the red line segment is bent and deformed due to the impact stress of the steel plate. We only consider the green dashed square frame. It can be seen from Figure 9b that the red line segment rotates and compresses due to two plastic hinges (black dots are plastic hinges). When the red line segment is deformed to the level, the deformation of this stage ends. The distance of the work done by the external force is $\Delta x=\sqrt{3}Nl/2$. $W_{ex}$ is used to represent the work done by the external force, therefore,

(9) $$W_{ex}=\frac{3Nlb}{2}\Delta x\sigma_{x}=\frac{3\sqrt{3}N^{2}l^{2}b}{4}\sigma_{x}$$

where $\sigma_{x}$ represents the uniform stress at the distal end.

Then, consider the rotation of the red line segment. As shown in Figure 9b, the plastic dissipation energy generated by the rotation of the two plastic hinges in the green dashed frame is $W_{h}=2M_{P}\Delta \theta$. The plastic dissipation torque of the macrostructure structure is $M_{p}=3bl^{2}\sigma_{0}/4$. Then, the plastic dissipation energy can be obtained as follows:

(10) $$W_{h}=\frac{\pi l^{2}b}{2}\sigma_{0}$$

where $\sigma_{0}$ is the static failure stress of the ADAH, as shown in Equation (8). Therefore, we must first derive the static failure stress of the ADAH element.

It can be seen from Figure 9b that the red line segment rotates and also shortens, therefore, we also need to calculate the plastic dissipation energy of compression, to obtain the plastic dissipation energy in compression as follows:

(11) $$W_{d}=\frac{\sqrt{3}Nl^{2}b}{2}\sigma_{0}$$

According to work and energy conversion, we obtain $W_{ex}=W_{h}+W_{d}$ and, finally, we can deduce the quasi-static crushing stress of SHH in the x direction as:

(12) $$\sigma_{x}=\frac{2\left(\pi +\sqrt{3}N\right)}{3\sqrt{3}N^{2}}\sigma_{0}=\frac{2\left(\pi +\sqrt{3}N\right)\left(\pi +\theta_{2}\right)\sin^{2}\theta_{1}}{3\sqrt{3}N^{2}\sin\left(\theta_{1}-\theta_{2}\right)\left(2\sin\theta_{1}\sin\theta_{2}-\sin\theta_{2}\right)}{\left(\frac{h}{l}\right)}^{2}\sigma_{ys}$$

**Figure 10:**
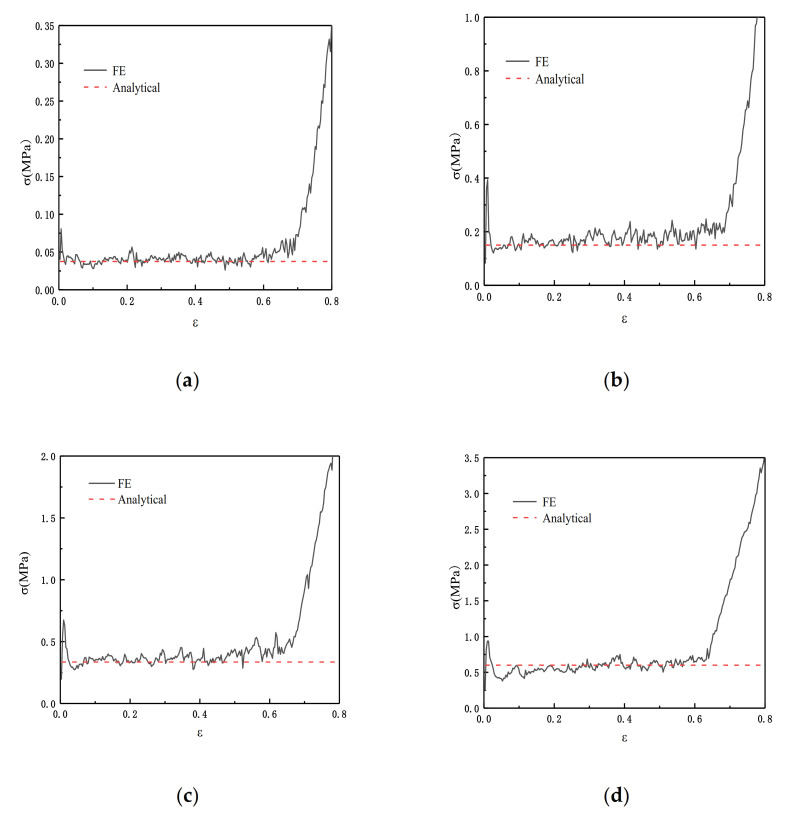
FE prediction of the quasi-static failure stress of SHH structure in the x direction, N=5. (**a**) $\bar{\rho }=2.5\%$; (**b**) $\bar{\rho }=5\%$; (**c**) $\bar{\rho }=7.5\%$; (**d**) $\bar{\rho }=10\%$.

Figure 10 shows the comparison between the FE prediction and the result calculated by Equation (12). $\theta_{1}=\pi /3$ and $\theta_{2}=\pi /6$ in Equation (12).

### 3.3. Uniaxial Compression in the z Direction

**Figure 11:**
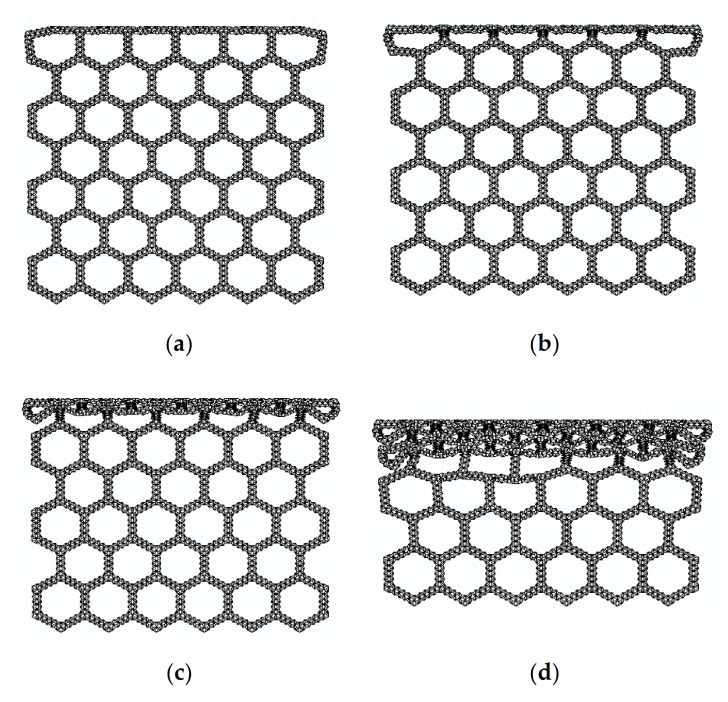
FE predicting quasi-static compression in the z direction, N=5, and $\bar{\rho }=2.5\%$. (**a**) ε=5%; (**b**) ε=10%; (**c**) ε=20%; (**d**) ε=40%.

**Figure 12:**
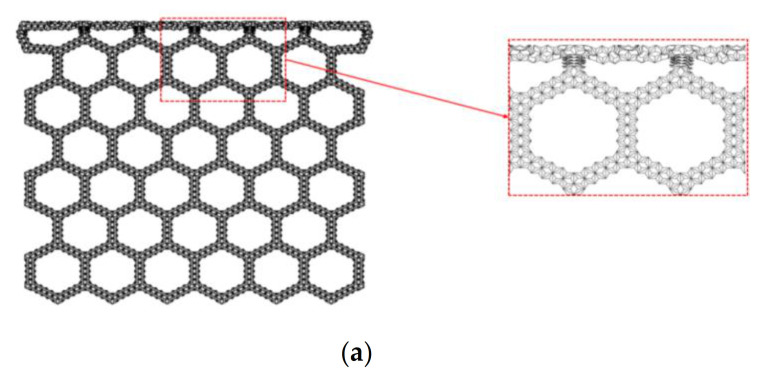

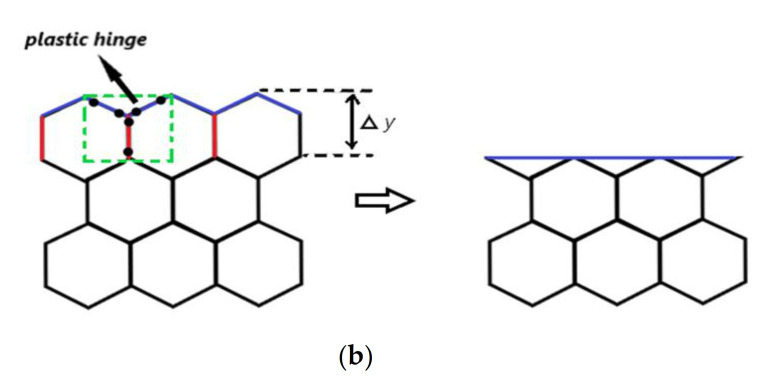
(**a**) Enlarged SHH deformation diagram, N=5, ε=10%, and $\bar{\rho }=2.5\%$; (**b**) diagram of macrostructure deformation, impact in the z direction.

In the previous section, we analyzed the static failure stress of the SHH in the x direction. In this section, we study the static failure stress of the SHH in the z direction. When the SHH is impacted in the x direction, first, the failure zone assumes an “X” shape, then, transforms into a “V” shape, and finally completes the first stage of deformation and enters the second stage. Unlike in the x direction, the SHH presents a layer-by-layer deformation mode when compressed in the z direction, as shown in Figure 11. In order to facilitate the analysis, we enlarge part of Figure 11a, and the result is shown in Figure 12a. Following the analysis in the previous section, we use a regular hexagon to represent the macro structure of the SHH. We only study the structure in the green box. When the rigid plate contacts the SHH, the blue line segment begins to deform due to the impact, and then begins to rotate and compress. After the blue line segment reaches the horizontal position, the red line segment begins to be compressed, and finally, is fully compressed. The red line segment also rotates while being compressed. On further loading, there is a similar deformation in the next stage. Please refer to the supplementary materials for the specific deformation mode.

According to the deformation principle in Figure 12b, we can derive an analytical model of the collapse stress of the SHH structure in the z direction. The derivation method is the same as that of the SHH in the x direction. First, the work done by the external force is as follows:

(13) $$W_{ex}=\frac{3\sqrt{3}N^{2}l^{2}b}{2}\sigma_{z}$$

where $\sigma_{z}$ represents the uniform stress applied, in the z direction, at the distal end.

It can be seen from Figure 12b that the plastic dissipation energy generated by the six plastic hinges is as follows:

(14) $$W_{h}=\frac{7\pi l^{2}b}{8}\sigma_{0}$$

In addition, the plastic compression energy needed in the compression of the red line segment and the blue line segment is as follows:

(15) $$W_{d}=\sqrt{3}\left(3-\sqrt{3}\right)Nl^{2}b\sigma_{0}$$

According to Equations (13)–(15), the static failure stress of SHH on shaft z can be obtained as:

(16) $$\sigma_{y}=\frac{7\pi +24N\left(\sqrt{3}-1\right)}{12\sqrt{3}N^{2}}\sigma_{0}=\frac{\left[7\pi +24N\left(\sqrt{3}-1\right)\right]\left(\pi +\theta_{2}\right)\sin^{2}\theta_{1}}{12\sqrt{3}N^{2}\sin\left(\theta_{1}-\theta_{2}\right)\left(2\sin\theta_{1}\sin\theta_{2}-\sin\theta_{2}\right)}{\left(\frac{h}{l}\right)}^{2}\sigma_{ys}$$

**Figure 13:**
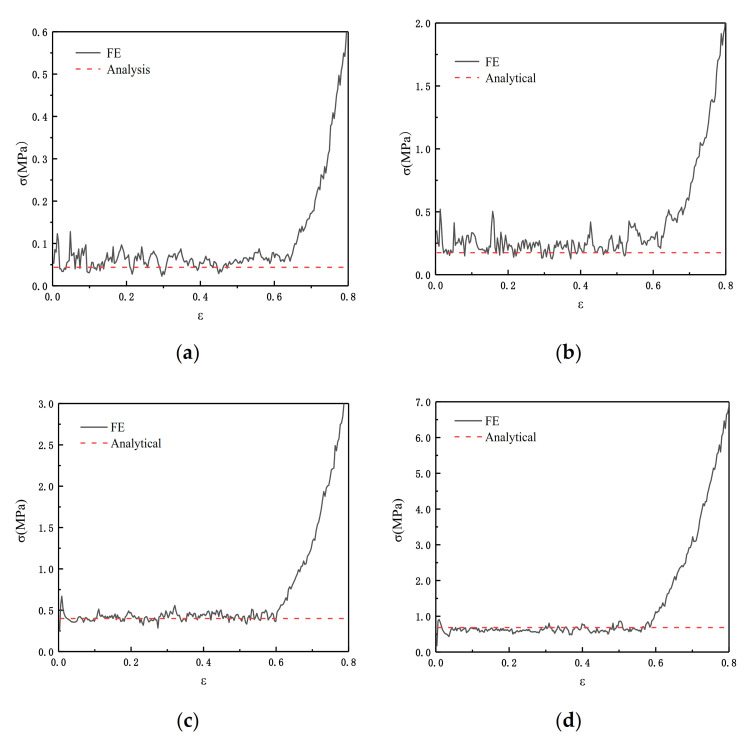
FE prediction of the static failure stress of SHH structure in the z direction, N=5. (**a**) $\bar{\rho }=2.5\%$; (**b**) $\bar{\rho }=5\%$; (**c**) $\bar{\rho }=7.5\%$; (**d**) $\bar{\rho }=10\%$.

From Figure 13 we can see the comparison result of Equation (16) and the FE. We carried out four groups of experiments with relative densities of 2.5%, 5%, 7.5%, and 10%, respectively. $\theta_{1}=\pi /3$ and $\theta_{2}=\pi /6$ in Equation (16). The results show that when the relative density is relatively small, Equation (16) has a better simulation effect.

### 3.4. Dynamic Damage Response

**Figure 14:**
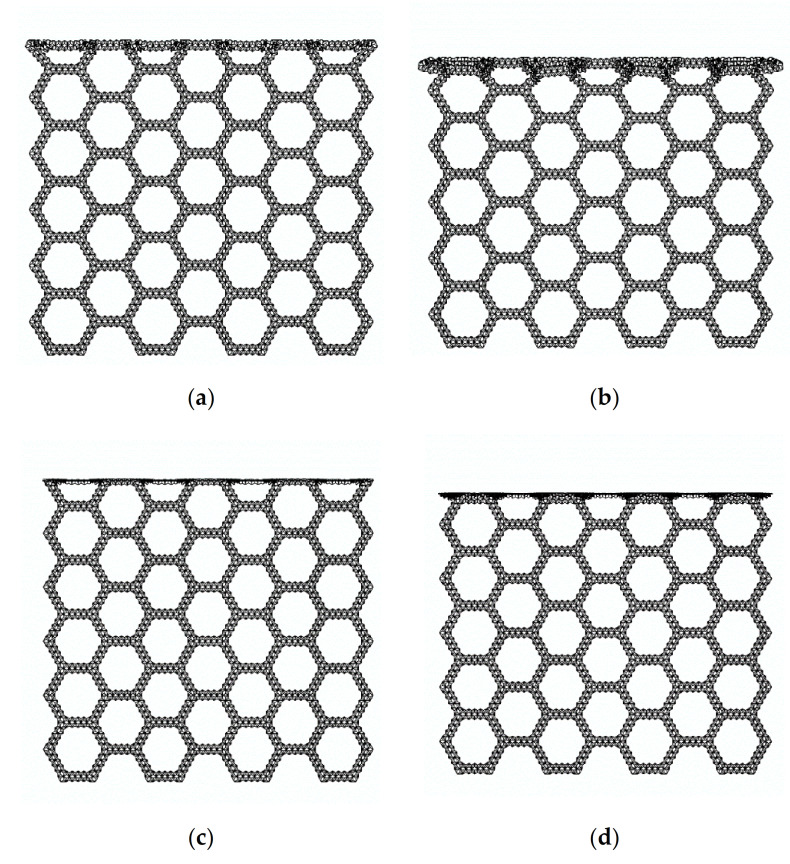
FE prediction of the SHH dynamic damage impact, the impact direction is x, N=5, and $\bar{\rho }=5\%$. (**a**) ε=10%, V=50 m/s; (**b**) ε=17%, V=50 m/s; (**c**) ε=10%,V=200 m/s; (**d**) ε=17%, V=200 m/s.

In this section, we study the dynamic damage response of the SHH. Unlike the static response, in both directions, the dynamic response has a deformation mode that destroys layer-by-layer. First, we study the dynamic damage response of SHH in the x direction. The quasi-static failure and deformation of SHH in the x direction, first, shows an “X” shaped structure. As the compression continues, when the fracture strain ε reaches 24%, the “X” structure begins to transform into a “V” shaped structure. The dynamic damage response is shown Figure 14a,b. It shows that when the speed is 50 m/s, the failure and deformation appear as a layer-by-layer mode. As the speed increases (as shown in Figure 14c,d, the impact speed is 200 m/s), this layer-by-layer destruction becomes more obvious. The corresponding form of the dynamic damage of SHH in the z direction is similar to that of the static, and the faster the speed the more obvious the deformation mode becomes. We can obtain the dynamic failure stress through the law of conservation of momentum [21]. For impulse we obtain the following:

(17) $$I=A\int_{0}^{t}\left(\sigma_{d}-\sigma_{2}\right)\;dt$$

where A is cross-sectional area of the element, $\sigma_{d}$ is the dynamic failure stress, $\sigma_{2}$ is the uniform stress at the distal end (quasi-static failure stress), and $t=\varepsilon_{d}H/V$ is the time it takes for the element to collapse to densification.

Here, we can obtain the expression of dense strain. The experiments show that when the speed increases to 200 m/s, the expression of compact strain is closer to $1-\bar{\rho }$, and therefore we obtain the following equation [22]:

(18) $$\varepsilon_{d}=\left\{\begin{array}{ll} \left(0.8+0.2V/V_{0}\right)\left(1-\bar{\rho }\right), & \;V\leq V_{0}\\ \left(1-\bar{\rho }\right), & \;V>V_{0} \end{array}\right.$$

where $V_{0}$ is a parameter that changes according to size and, in this study, $V_{0}=200\;m/s$.

The momentum change of the unit can be expressed as:

(19) $$\Delta P=AH\bar{\rho }\;\rho_{s}V$$

According to the law of conservation of momentum:

(20) I=ΔP

Therefore, according to Equations (17)–(20), we obtain the following:

(21) $$\sigma_{d}=\sigma_{x}+\rho_{s}\bar{\rho }\frac{V^{2}}{\varepsilon_{d}}$$

For the impact compression in the z direction, just change x to z.

**Figure 15:**
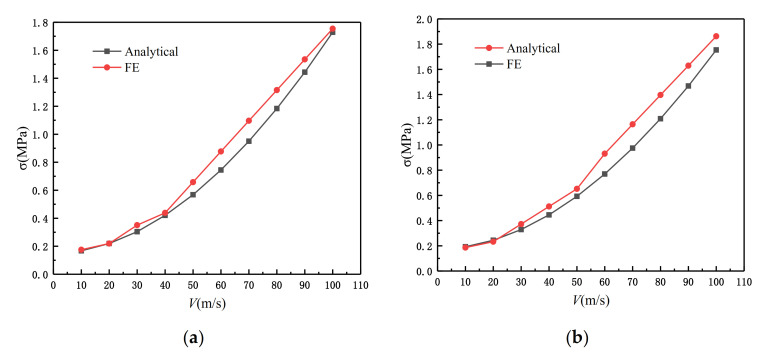
Dynamic failure stress of SHH at different speeds, $\bar{\rho }=5\%$ and N=5. (**a**) Impact in the x direction; (**b**) impact in the z direction.

**Figure 16:**
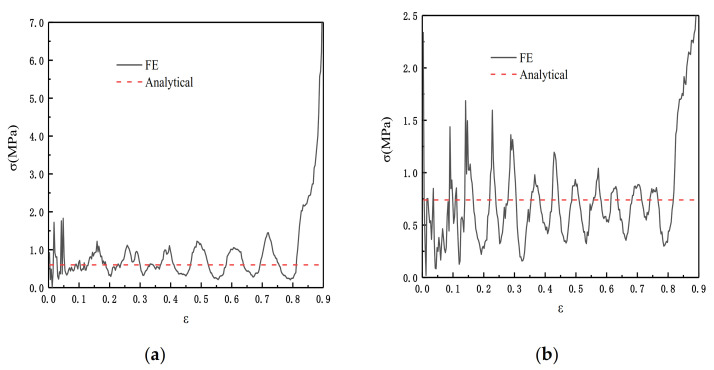
Dynamic failure stress of SHH, V=50 m/s, $\bar{\rho }=5\%$, and N=5. (**a**) Impact in the x direction; (**b**) impact in the z direction.

According to Equation (21), we can carry out the finite element simulation and comparison. We set the impact velocity gradient from 10 to 100 m/s. $\theta_{1}=\pi /3$ and $\theta_{2}=\pi /6$. The simulation result is shown in Figure 15, and the error between the experimental data and the fitting result of Equation (21) is within the acceptable range. To facilitate the comparison, we show the simulation diagram when the speed is 50 m/s. From Figure 16, it can be seen that whether or not it is an impact in the x or z directions, the shock and fluctuation of the SHH crushing stress are both large due to inertia. This is because in the case of quasi-static crushing and low-speed impact, the substructure plays a leading role in the slip stress, while in high-speed loading, the inertia effect dominates.

### 3.5. The Influence of the Number of Substructures on the SHH Failure Stress

**Figure 17:**
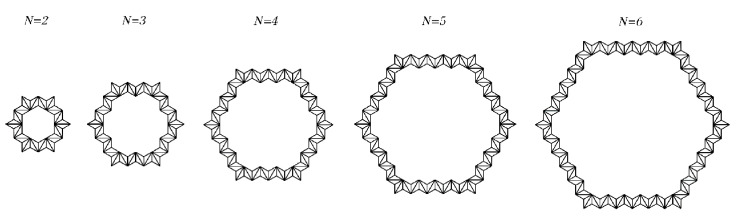
Schematic diagram of ADAH structure when N=2∼6

**Figure 18:**
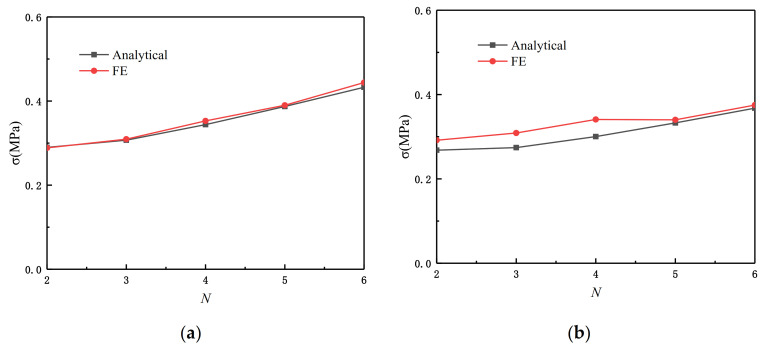
The quasi-static failure stress of SHH under uniaxial compression, N=2∼6 and $\bar{\rho }=7.5\%$. (**a**) Impact in the x direction; (**b**) impact in the z direction.

For the derivation of the previous Equation (12) and Equation (16), we only compare the experimental data when N=5. In this section, we change N and set the length to 5 and the interval to 1. The values of $\theta_{1}$ and $\theta_{2}$ are consistent with those of $\theta_{1}$ and $\theta_{2}$ when N=5. We explore Equation (12) and Equation (16) and compare the finite element analysis results when N=2∼6. Figure 17 is a schematic diagram of the SHH substructure when N=2∼6. We set the relative density $\bar{\rho }=7.5\%$ and study the static failure stress of the SHH in the x and z directions. The various settings of the finite element are the same as when N=5, and the comparison of the results is shown in Figure 18. From the results, the theoretical result of Equation (21) demonstrates that, for both directions of applied impact, the resulting static failure stress increases as N increases. According to the finite element analysis, due to different microstructures, the results are slightly different, but the error is within the acceptable range. We can see from Figure 18a that the data fit well. This is because when N changes, the deformation mode in the x direction is still roughly the same, so the fitting is ideal. For the data fitting in the z direction, due to the slight difference in the deformation mode, the data also have slight differences but are also within the acceptable range. For the numerical prediction results of the collapse stress in the x and z directions of the model with N=2∼6, there are local optimal results when N=5 and 6. The predicted collapse stress increases monotonically with N. However, a nearly monotonic increase in N is shown, which is due to the fact that they are based upon simplified failure modes. Nevertheless, the overall agreement between numerical forecasts and analytical forecasts is acceptable.

## 4. Conclusions

In this study, we conducted a numerical analysis of the static and dynamic failure of an SHH structure. When N=5, the static and dynamic equation derivations of the SHH under uniaxial compression in both x and z directions are explored and are compared with the finite element method. The SHH performance is superior to the FHH and ADAH during compression, and as N increases, the performance superiority of the SHH becomes more obvious.

During the static failure analysis of the SHH, we found that in quasi-static crushing, the layered design of the SHH significantly improved the collapse stress of the FHH and ADAH. When the impact direction was x, the deformation mode changed from “X” shaped to “V” shaped, and then proceeded to the next stage of deformation. When the impact direction was z, it showed a layer-by-layer destruction. When the SHH was subjected to dynamic failure impact, the fitting results of Equation (21) at different speeds showed that as the speed increased, the dynamic damage stress also increased, and in both directions the impact always had a layer-by-layer damage effect. The greater the speed, the more obvious the layer-by-layer destruction effect. This is because, in the case of quasi-static crushing and low-speed impact, the substructure plays a leading role in the slip stress, while in high-speed loading, the inertial effect dominates and the influence of structural design can be ignored. Finally, by studying the influence of different N values on the SHH structure, we conclude that the error between theoretical analysis and numerical calculation fitting is within an acceptable range.

## Data Availability

The data presented in this study are available on request from the corresponding author. The data are not publicly available due to programming privacy in structural design.

## Supplementary Materials

The following are available online at https://www.mdpi.com/article/10.3390/ma14081917/s1, Video S1: SHH deformation in direction *x*; Video S2: SHH deformation in direction *z*.
